# Label-Free Ag^+^ Detection by Enhancing DNA Sensitized Tb^3+^ Luminescence

**DOI:** 10.3390/s16091370

**Published:** 2016-08-26

**Authors:** Kimberly Kleinke, Runjhun Saran, Juewen Liu

**Affiliations:** Department of Chemistry and Waterloo Institute for Nanotechnology, University of Waterloo, Waterloo, ON N2L 3G1, Canada; kkleinke@uwaterloo.ca (K.K.); r.saran.bhatnagar@gmail.com (R.S.)

**Keywords:** aptamers, biosensors, fluorescence, luminescence, terbium, silver

## Abstract

In this work, the effect of Ag^+^ on DNA sensitized Tb^3+^ luminescence was studied initially using the Ag^+^-specific RNA-cleaving DNAzyme, Ag10c. While we expected to observe luminescence quenching by Ag^+^, a significant enhancement was produced. Based on this observation, simple DNA oligonucleotide homopolymers were used with systematically varied sequence and length. We discovered that both poly-G and poly-T DNA have a significant emission enhancement by Ag^+^, while the absolute intensity is stronger with the poly-G DNA, indicating that a G-quadruplex DNA is not required for this enhancement. Using the optimized length of the G7 DNA (an oligo constituted with seven guanines), Ag^+^ was measured with a detection limit of 57.6 nM. The signaling kinetics, G7 DNA conformation, and the binding affinity of Tb^3+^ to the DNA in the presence or absence of Ag^+^ are also studied to reveal the mechanism of emission enhancement. This observation is useful not only for label-free detection of Ag^+^, but also interesting for the rational design of new biosensors using Tb^3+^ luminescence.

## 1. Introduction

Silver is a very common coinage metal and it has been used for a diverse range of applications in electronics, catalysis, spectroscopy and biomedical research [1]. Exposure to a high level of Ag^+^ causes a number of adverse health effects to the skin, eyes and many internal organs [2]. Measurement of silver is needed for environmental monitoring and for recovery of this metal from mining and industrial waste. In addition to relying on analytical instruments such as ICP-MS, portable sensors are attractive for on-site and real-time detection. A few small molecule based Ag^+^ sensors have been reported [3,4]. We are interested in developing DNA-based biosensors because of their high stability, low cost and programmability [5,6,7,8,9,10].

The interaction between DNA and Ag^+^ is an interesting and important topic from a few different perspectives. Some early work in this regard involved DNA-templated Ag^+^ reduction and silver nanowire formation [11,12,13], where the interaction is believed to be mainly electrostatically driven and non-specific. Since 2004 [14], cytosine-rich DNA and at some instances guanine-rich DNA have been used for preparing fluorescent silver nanoclusters [14,15,16,17,18]. Ag^+^ has a strong and quite specific affinity for DNA pyrimidine bases, in particular, cytosines [19,20,21], and Ag^+^ can stabilize C-Ag^+^-C base pairs. This knowledge has been used to design biosensors based on DNA folding for Ag^+^ detection [22,23]. DNAzymes are DNA-based catalysts that often recruit metal ions. We recently reported an Ag^+^-specific RNA-cleaving DNAzyme named Ag10c [24]. Ag10c cleaves its substrate only in the presence of Ag^+^, thus allowing highly selective detection of silver down to 25 nM Ag^+^.

Sensitized Tb^3+^ luminescence is a powerful method for probing the structure and folding of nucleic acids [25,26,27]. Free Tb^3+^ ions are very weakly luminescent due to their low extinction coefficient. When bound with DNA, especially guanine, its emission intensity is drastically increased due to energy transfer from DNA. With a specific ligand competitively binding to the Tb^3+^ bound DNA, the Tb^3+^ luminescence might decrease due to competition and subsequent replacement by this ligand. Using this method, we recently studied the binding strength of lanthanides to DNA [28], and binding of Na^+^ by another DNAzyme [27,29]. In this work, we initially aimed to use Tb^3+^ luminescence to study Ag^+^ binding to the Ag10c DNAzyme. Instead of the expected fluorescence decrease, we found a very large fluorescence enhancement in the presence of higher concentrations of Ag^+^. This has led us to systematically explore this system, leading to a highly sensitive and label-free detection of Ag^+^ using poly-G DNA.

## 2. Materials and Methods

**Chemicals.** All the DNA samples were from Eurofins (Huntsville, AL, USA). Silver nitrate, terbium chloride and other metal salts were purchased from Sigma-Aldrich (St. Louis, MO, USA) at the highest available purity. Tris(hydroxymethyl)aminomethane (Tris), 2-(*N*-morpholino)ethanesulfonic acid (MES), and 2-[4-(2-hydroxyethyl)piperazin-1-yl]ethanesulfonic acid (HEPES) were from Mandel Scientific Inc. (Guelph, ON, Canada).

**Fluorescence spectroscopy.** All the Tb^3+^ luminescence spectra were collected using a Fluoromax 4 fluorometer by exciting at 290 nm. Unless otherwise indicated, the DNA concentration was 1 µM for all the experiments, and the Tb^3+^ concentration was 5 µM. The buffer has been optimized and most experiments were performed in HEPES buffer (pH 7.5).

**CD spectroscopy.** CD spectra were obtained using a J-715 spectropolarimeter (JASCO, Easton, MD, USA) at 25 °C. The final G7 concentrations was 1 µM. Each measurement was recorded between 320 to 220 nm at a scanning rate of 100 nm·min^−1^ using a sealed 1-mm path length quartz cuvette. The spectra were collected with a response time of 1 s. The final spectra were the averages of ten measurements. The cell holding chamber was flushed with a constant stream of dry nitrogen gas to avoid water condensation on the cell exterior.

## 3. Results and Discussion

### 3.1. Enhanced Emission with the Ag10c DNAzyme

**Figure 1:**
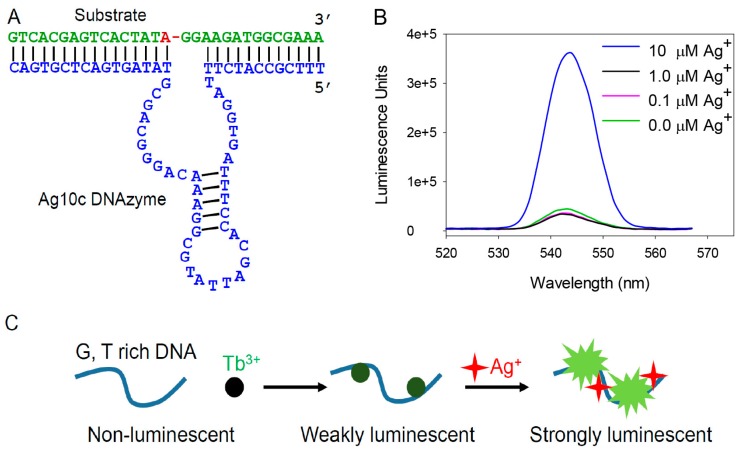
(**A**) The secondary structure of the Ag10c DNAzyme. For the Tb^3+^ luminescence experiment, the substrate is the all-DNA analog and cannot be cleaved; (**B**) Tb^3+^ luminescence spectroscopy with the Ag10c DNAzyme in the presence of 0, 0.1, 1 and 10 µM concentrations of Ag^+^; (**C**) A scheme showing the effect of Ag^+^ in promoting the luminescence of poly G, T DNA.

The secondary structure of the Ag10c DNAzyme is shown in Figure 1A [24]. This DNAzyme was originally obtained by in vitro selection performed in the presence of Ag^+^ and it is highly specific for Ag^+^. It is composed of a substrate strand and an enzyme strand. The original substrate contains a single RNA linkage serving as the cleavage site (the red adenine position). The enzyme strand (in blue) has a hairpin and two loops. In the presence of Ag^+^, the substrate is cleaved into two pieces by the enzyme strand. In this work, we are interested in studying silver binding instead of cleavage. To avoid cleavage, we used a full-DNA analog of the substrate so that we probe only Ag^+^ binding.

We observed a weak luminescence peak at 542 nm when the DNAzyme was mixed with Tb^3+^ (Figure 1B, green curve), while the same concentration of Tb^3+^ without the DNAzyme was essentially non-luminescent (data not shown). With 0.1 or 1 µM Ag^+^, a very small amount of fluorescence quenching was observed. We expect that Ag^+^ can specifically bind to the DNAzyme (since this DNAzyme is specifically activated by Ag^+^), thus we speculated that this would cause weakening of Tb^3+^ binding and thus a decrease in its emission [29]. While the decreased emission did occur at low Ag^+^ concentrations, the amount of signal change was extremely small (<10%). When 10 µM Ag^+^ was added, surprisingly, instead of luminescence quenching, nearly 8-fold enhancement was observed (Figure 1B, blue curve). Therefore, the net emission change might be due to both Ag^+^-induced Tb^3+^ displacement (e.g., quenching), and Ag^+^-induced Tb^3+^ emission enhancement. With a higher Ag^+^ concentration, the enhancement effect dominated. Therefore, Tb^3+^ luminescence cannot be used to study the specific binding of Ag^+^ to the Ag10c DNAzyme due to the complication of data interpretation. On the other hand, it appears to be an interesting system for developing a label-free method for Ag^+^ detection.

### 3.2. Effect of Ag^+^ with DNA Homopolymers

Such a strong Ag^+^ induced emission enhancement indicates the possibility of developing an Ag^+^ sensor. The Ag10c DNAzyme itself, however, is not sensitive enough since it requires 10 µM Ag^+^ to achieve a strong emission enhancement. By searching the literature, we found that silver and DNA (or nucleotides) can produce or influence fluorescence in many different ways. For example, Ag^+^ coordinates with 9-allyladenine and produces a fluorescent product with an emission peak at 410 nm [30]. DNA can also template the formation of fluorescent silver nanoclusters [14]. Since the emission peak we observed is from Tb^3+^, and we did not add any reducing agent, it is unlikely that our observations were related to silver nanoclusters. In regard to the sensitized lanthanide luminescence, silver nanoparticles were reported to enhance the emission of a Eu^3+^ complex [31]. Adenosine monophosphate (AMP) and Tb^3+^ also can form weakly luminescent complexes and the emission is known to get enhanced by 8-fold by adding Ag^+^. This scheme allows a detection limit of 60 nM Ag^+^ [32].

**Figure 2:**
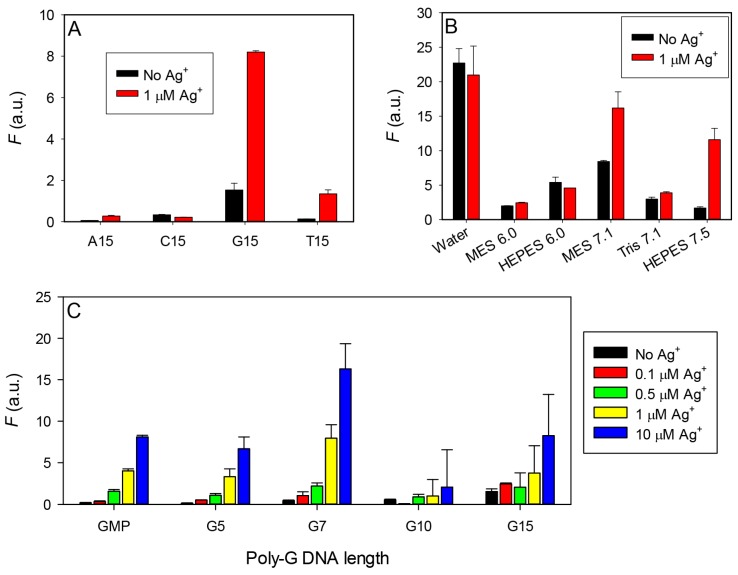
Effect of the (**A**) different 15-mer DNA; (**B**) buffer type and pH; and (**C**) different poly-G DNA length on the sensitivity of Ag^+^ in promoting Tb^3+^ luminescence. These experiments used 1 µM DNA and 5 µM Tb^3+^. The buffer contained 50 mM HEPES buffer, pH 7.5 except for that in (**B**). The luminescence intensities were collected by exciting at 290 nm and monitoring emission at 543 nm.

To have a full understanding, we next tested different 15-mer DNA sequences (Figure 2A). Overall, A15 and C15 gave very weak luminescence regardless of Ag^+^ concentration. Both G15 and T15 showed Ag^+^-enhanced emission. The overall intensity was much higher with the G15. Therefore, we focused on poly-G DNA for the subsequent studies. Based on these results, we also conclude that the observed emission enhancement in the Ag10c DNAzyme (Figure 1B) is likely due to its nucleotides instead of the specific Ag^+^ binding.

Before further optimization of DNA sequence, we next studied the effect of buffer composition and pH using the G15 DNA (Figure 2B). In the Tris buffer, regardless of the tested pH, no fluorescence change was observed. This can be attributed to the fact that Tris is a coordination buffer with a free primary amine and it may sequester the Ag^+^ and Tb^3+^ ions. MES buffer has a pK_a_ value of 6.2 and we tested the response at pH 6.0 and 7.1. A better response was observed at higher pH. Next we tested the HEPES buffer whose pK_a_ is 7.6, allowing us to study higher pH. In this case, we observed the best response at pH 7.5. Finally, we wanted to see how this system behaves in just water without buffer. We observed negligible luminescence change upon addition of Ag^+^, which is consistent with its relatively acidic pH of ~6. Overall, a non-coordinating buffer with a higher pH appears to give a larger emission change. We did not try even higher pH to avoid precipitation of Ag^+^ and Tb^3+^ and decided to do all the further experiments in 50 mM HEPES pH 7.5.

**Figure 3:**
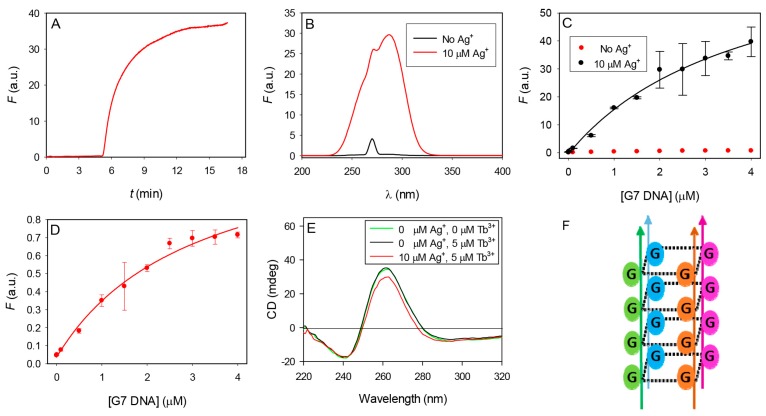
Mechanistic studies. (**A**) Kinetics of emission change upon addition of 10 µM Ag^+^ at 5 min; (**B**) Fluorescence excitation spectra of Tb^3+^ (5 µM) and G7 (1 µM) in the absence and presence of 10 µM Ag^+^ in 50 mM HEPES pH 7.5 buffer by monitoring emission at 543 nm; (**C**) Titration of G7 to Tb^3+^ in the absence and presence of Ag^+^; (**D**) The red trace in (**C**) at a smaller *y*-axis scale to determine the *K*_d_ values based on *F* = *F*_0_ + *aC*/(*K*_d_ + *C*), where *F* and *F_0_* are the current and initial fluorescence intensity, respectively, *C* is added DNA concentration, and *a* is the final fluorescence enhancement at infinitely high DNA concentration; (**E**) The CD spectra of 1 µM G7 DNA in 50 mM HEPES pH 7.5 and upon the sequential addition of 5 µM Tb^3+^ and then 10 µM Ag^+^; (**F**) A general schematic of the four-stranded parallel G-quadruplex structure.

We next tested the poly-G DNA of different lengths (G15, G10, G7, G5, 1 µM each) and GMP (10 µM) respectively (Figure 3C). For each DNA, various concentrations of Ag^+^ were titrated. Interestingly, the G7 DNA showed the overall highest sensitivity and signal. With 10 µM Ag^+^, the Tb^3+^ emission enhanced by 17-fold. DNA longer than that had a much lower signal increase by Ag^+^ due to a high background. The G5 DNA had only about half of the signal. Free GMP, although used at 10 µM, produced a signal similar to that from 1 µM of the G5 DNA. In a previous paper, it was reported that AMP is able to produce Ag^+^ induced fluorescence enhancement, with the best increase being only 8-fold [32]. We found that the G7 DNA works the best in this study with a much larger signal enhancement.

### 3.3. Mechanism Studies

We noticed that for a long DNA such as G15, the sample became cloudy as Ag^+^ was titrated in the presence of Tb^3+^. This suggests formation of a ternary complex with DNA, Tb^3+^ and Ag^+^. In other words, Ag^+^ might exert its function by binding to the DNA/Tb^3+^ complex. Shorter DNA such as G7 did not precipitate and this might be the reason that G7 worked better than G15. If this is the case, we predict that Ag^+^ signaling might be a kinetically slow process. To test this, we monitored the background fluorescence of the G7/Tb^3+^ complex for 5 min and then added 10 µM Ag^+^ (Figure 3A). Indeed, the emission gradually increased and it took more than 5 min for the signal to reach a relatively stable value. Therefore, in this study, we waited 5 min before measuring the emission intensity to obtain consistent results.

We also measured the fluorescence excitation spectra of the G7/Tb^3+^ complex in the absence and presence of 10 µM Ag^+^ (Figure 3B). With Ag^+^, the peak is much stronger, but overall, it has the feature of guanine absorption spectrum. Therefore, Ag^+^ has enhanced the energy transfer efficiency from DNA to Tb^3+^. One possibility is that Ag^+^ might have changed the binding stability of DNA. To measure the binding affinity, we fixed the Tb^3+^ concentration and titrated DNA in the absence or presence of Ag^+^ (Figure 3C). While in the presence of Ag^+^, the overall emission intensity is much higher (black curve), its dissociation constant (*K*_d_) was determined to be 3.9 ± 1.2 µM G7 DNA and is very similar to that in the absence of Ag^+^ (3.1 ± 0.8 µM). Therefore, Ag^+^ did not really affect the stability of binding between Tb^3+^ and DNA.

Based on the above studies, we propose that to promote Tb^3+^ emission, Tb^3+^ needs to be associated with DNA via optimal binding site(s). Without Ag^+^, the binding is random, while Ag^+^ can selectively occupy certain binding sites in DNA and thus forcing Tb^3+^ to be associated with other sites that are more efficient for energy transfer. For example, the binding between GMP and Ag^+^ has been carefully studied by Petty and co-workers [33]. They proposed that Ag^+^ can induce stacking of GMP via N7 and O6 coordination.

Zhang et al. also reported an Ag^+^-based enhancement, but in that case, a duplex made of [G3T]_5_ and its cDNA was formed initially [22,34]. Its cDNA was rich in cytosine and could form C-Ag^+^-C structures. Since the duplex DNA cannot promote Tb^3+^ emission, the enhancement effect was proposed to be due to the Ag^+^-induced removal of the cDNA. However, in our case, Ag^+^ directly participates in the luminescence enhancement due to formation of a ternary complex. In their system, the [G3T]_5_ DNA was used since it has the highest effect in promoting Tb^3+^ luminescence. Here we used only the G7 DNA without its cDNA, and the emission enhancement is from the effect of Ag^+^ binding to the same DNA to which Tb^3+^ binds.

Usage of transition metals such as Zn^2+^ to enhance the lanthanide luminescence intensity was also reported in other systems unrelated to DNA [35]. The choice of metal ion appears to be related to the coordination chemistry of the ligand, and in case of DNA, Ag^+^ appears to be quite an efficient metal ion for this purpose. In a previous study, upon using another tetra(amino acid) ligand, Ag^+^ produced enhancement of lanthanide emission which was attributable to a more rigid structure formation by Ag^+^ coordination [36].

Recently, Tan et al. used a 21-mer G-quadruplex forming DNA with the following sequence: GGGTTAGGGTTAGGGTTAGGG. They proposed that Tb^3+^ can stabilize the G-quadruplex structure, but the whole complex is poorly luminescent. Ag^+^ can disrupt the quadruplex structure enhancing the emission intensity [37]. This is very similar to our observation. However, our Ag10c DNAzyme cannot form the G4 structure, and even poly-T DNA can achieve quite a strong enhancement. Therefore, a special *G*-quadruplex forming DNA is not necessary in our scheme. Since we focused on the G7 DNA in this study, to test if the luminescence enhancement is related to the disruption of its tertiary structure upon addition of Ag^+^, we used circular dichroism (CD) spectroscopy to follow the reactions. We first measured the CD spectrum of 1 µM G7 DNA in 50 mM HEPES pH 7.5 and observed a positive peak at ~260 nm suggesting a four-stranded parallel *G*-quadruplex structure (Figure 3E,F). No change was observed upon the addition of 5 µM Tb^3+^ and only a slight decrease in the intensity occurred upon further addition of 10 µM Ag^+^. Therefore, these metal ions did not perturb the structure of the G7 DNA significantly.

### 3.4. Sensor Performance

**Figure 4:**
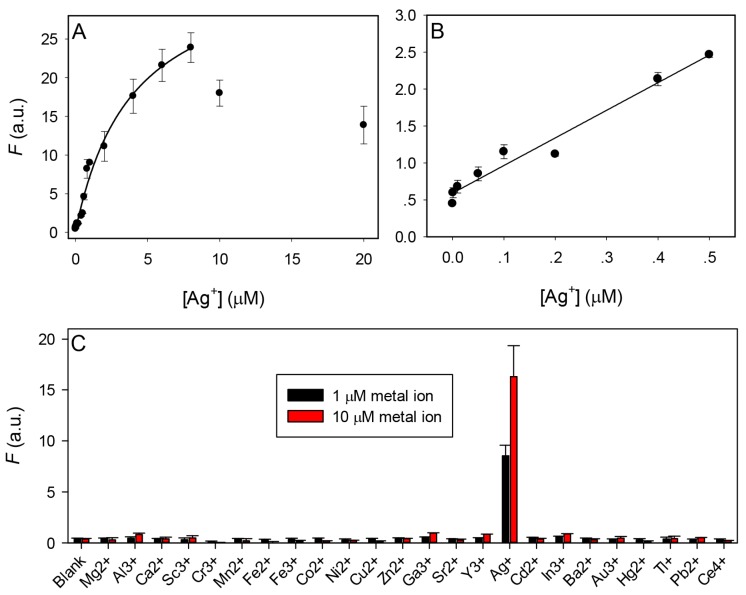
The performance of the G7 DNA (1 µM) and Tb^3+^ (5 µM) based sensor for Ag^+^ detection in 50 mM HEPES pH 7.5 buffer. (**A**) Sensor response to various Ag^+^ concentrations; (**B**) The sensor response at low Ag^+^ concentrations; (**C**) Sensor selectivity test at two metal concentrations. Only Ag^+^ produced the signal. Each experiment in this paper was repeated at least twice and the error bars represent the standard deviation of these measurements.

With the excellent fluorescence enhancement from the G7 DNA, we next measured its performance as a biosensor for Ag^+^ detection using the scheme shown in Figure 1C. First, Ag^+^ was carefully titrated in the mixture of G7 and Tb^3+^, the Tb^3+^ emission intensity initially increased drastically reaching saturation when 8 µM Ag^+^ was added (Figure 4A). It is likely that Ag^+^ has also a quenching effect as a heavy metal. Between 0 and 0.5 µM Ag^+^, a roughly linear relationship between Ag^+^ concentration and the emission intensity can be found (*R*^2^ = 0.97, Figure 4B). Based on this slope, we calculate the detection limit to be 57.6 nM Ag^+^ by applying the formula 3 σ/slope, where σ is the standard deviation of the background variation in the absence of Ag^+^. This value is much lower than the maximum allowable contamination level of silver in water 0.1 mg/L (or 930 nM) defined by the World Health Organization. The selectivity was next tested at two different metal concentrations (1 and 10 µM) and Ag^+^ is the only metal that produced the signal enhancement (Figure 4C), indicating a very high selectivity. This sensing mechanism is attractive since it is label-free and has a large signal enhancement.

## 4. Conclusions

In this study, we initially intended to measure Ag^+^ binding by the Ag10c DNAzyme based on sensitized Tb^3+^ luminescence, where we expected to observe emission quenching. The unexpected but strong fluorescence enhancement observed however, led to the current study of using this system for Ag^+^ detection. We have systematically optimized the sequence and length of DNA. We found that poly-G DNA produces both a high relative enhancement over background and also a high absolute emission intensity. Using the optimized G7 DNA, we obtained a detection limit of 57.6 nM Ag^+^ with excellent selectivity. Since DNA is programmable, a short fragment of poly-G DNA might be incorporated into other DNA sequences for sensing other analytes beyond Ag^+^.
